# Provision of smoking cessation support for patients following a diagnosis of cancer in Ireland

**DOI:** 10.1016/j.pmedr.2023.102158

**Published:** 2023-02-20

**Authors:** P. Fitzpatrick, N. Bhardwaj, M. Masalkhi, A. Lyons, K. Frazer, A. McCann, S. Syed, V. Niranjan, C.C. Kelleher, S. Brennan, P. Kavanagh, P. Fox

**Affiliations:** a Dept. of Preventive Medicine and Health Promotion, St Vincent’s University Hospital, D04 T6F4, Elm Park Dublin 4, Ireland; b School of Public Health, Physiotherapy and Sports Science, University College Dublin, Belfield Dublin 4, Ireland; c School of Medicine, University College Dublin, Belfield Dublin 4, Ireland; d School of Nursing, Midwifery and Health Systems, Health Sciences Centre, University College Dublin, Belfield Dublin 4, Ireland; e UCD Conway Institute of Biomolecular and Biomedical Research and UCD School of Medicine, Ireland; f College of Health and Agricultural Science (CHAS), University College Dublin, Belfield Dublin 4, Ireland; g St Luke’s Hospital, Rathgar Dublin 6, Ireland; h Health Service Executive Tobacco Free Ireland Programme, Strategy and Research, 4th Floor, Jervis House, Jervis Street, Dublin 1, D01 W596, Ireland

**Keywords:** Smoking cessation, Cancer, Hospital, Cancer care, Oncology

## Abstract

There is growing evidence that smoking cessation (SC) improves outcomes following diagnosis of cancer. Notwithstanding adverse outcomes, a significant number of those diagnosed with cancer continue to smoke. Our objective was to document the SC services provided for patients with cancer by specialist adult cancer hospitals across Ireland, a country with a stated tobacco endgame goal.

A cross-sectional survey based on recent national clinical guidelines was used to determine SC care delivery across eight adult cancer specialist hospitals, and one specialist radiotherapy centre. Qualtrics was used.

The response rate was 88.9% with data reported from seven cancer hospitals and one specialist radiotherapy centre, all indicating they had some SC related provision (100%). Stop smoking medications were provided to cancer inpatients in two hospitals, at outpatients and attending day ward services in one hospital. Smokers with cancer were referred automatically to the SC service in two hospitals at diagnosis. While stop smoking medications were available 24 h a day in five hospitals, most did not stock all three (Nicotine Replacement Therapy, Bupropion, Varenicline). One hospital advised they had data on uptake of SC services for smokers with cancer but were unable to provide detail.

There is considerable variation in SC information and services provided to cancer patients across adult cancer specialist centres in Ireland, reflecting the suboptimal practice of smoking cessation for patients with cancer found in the limited international audits. Such audits are essential to demonstrate service gaps and provide a baseline for service improvement.

## 1 Introduction

Tobacco smoking is not only a cause of many cancers (Gandini et al., 2008); but for those who smoke and develop cancer, it is associated with poorer treatment outcomes (including treatment-related complications and toxicities), increased risk of recurrence (Florou et al., 2014), development of second primaries (Smith et al., 2019), lower survival, and decreased quality of life among continuing smokers (Florou et al., 2014). There is a growing body of evidence that smoking cessation (SC) improves outcomes following diagnosis of a number of cancers (Romaszko-Wojtowicz et al., 2018).

Unfortunately, approximately 1 in 5 smokers who have been diagnosed with cancer continue to smoke (Swoboda et al., 2019). Those who continue to smoke are more likely to be younger, less educated, uninsured, of marital status other than married or widowed, and to have survived cervical cancer (Wells et al., 2017). While the benefits of smoking cessation are well-established, and evidence of what constitutes effective stop smoking care is clear, many smokers using health services do not experience quality preventive care in this critical area (Papadakis et al., 2018, Evans et al., 2017). Wells *et al.* reported that only a small number of patients and families in the United Kingdom (UK) used the available National Health Service (NHS) SC services, with the majority indicating limited discussions related to SC with their oncology healthcare professionals (HCPs), although they would be agreeable to such discussions (Wells et al., 2017). Barriers to discussions around smoking cessation from the perspective of oncology HCPs include concerns regarding negative impact on the therapeutic relationship and inadequate training (Wells et al., 2017, Warren et al., 2013).

Our previous research in St Vincent’s University Hospital, Ireland, identified a majority of smokers were asked about smoking behaviours; 50% received advice and 51% identified as ‘*wanting to quit’*. However, 35% were offered Nicotine Replacement Therapy (NRT) and only 25% provided with an opportunity to speak to a smoking cessation practitioner; in addition, there were few referrals from oncology to smoking cessation services (Fitzpatrick et al., 2014).

Optimal SC services include training of all HCPs, identification of smokers pre or at admission, brief intervention, availability of specialist SC support services and medication, referral to SC support services, provision of one to one advice or group intervention with follow up to one year (Neubeck, 2006). Ireland has recently published the National Clinical Guidelines for smoking cessation, which recommends that every patient/service user who engages with frontline HCPs, should be asked about their smoking status, the response should be documented, and every smoker should be advised to quit and be offered support at every opportunity (Department of Health, 2022). Hospitals are ideal settings to deliver smoking cessation interventions as admission may trigger a cue to action (Department of Health, 2022). However, patients with a new diagnosis of cancer or undergoing cancer treatment may not fully fit this profile, with high levels of anxiety and worry predominating. Hospital campuses are supportive of quitting because they have been smoke-free since at least 2015 (HSE, n.d.) and any hospital visits to a smoke-free campus provide important opportunities for HCPs to promote SC (HIQA, 2017).

Although there are many studies looking at ways of managing smoking cessation in patients with cancer there have been no studies to date documenting the SC services provided by specialist cancer hospitals in Ireland for patients with cancer, in addition to very limited published international studies (Frazer et al., 2022). The aim of our study was to document existing SC services for patients with cancer specifically provided by the eight specialist cancer hospitals and one specialist radiotherapy hospital in the context of recently developed National Clinical Guidelines (circulated for consultation 2021, published 2022) (Department of Health, 2022).

## 2 Methods

An audit survey was developed using standards outlined in the ‘Stop Smoking *National Clinical Guidelines No. 28 Health Services Executive (HSE)*’ (Section 3: Recommendations 1-3,) (Department of Health, 2022) and ‘*Global Network Self-Audit Questionnaire and Planning Template*’, Standard 4 (Global Network for Tobacco Free Healthcare Services, n.d.). The survey sought to document the provision of SC advice, SC service referral and pharmacotherapy provision. The survey questions were based on the criteria for smoking cessation support provision as set out in the recently published national clinical guidelines for smoking cessation in Ireland (Department of Health, 2022).

In Ireland, all publicly provided adult cancer care is led by eight specialist centres and there is one publicly funded radiation oncology network. All eight specialist cancer centres are part of a tertiary referral university hospital, with the full range of medical and surgical specialties. All patients aged 16 and over diagnosed with cancer in the country are treated at one of these hospitals. No oncology services exist outside these hospitals. The specialist radiotherapy hospital is a standalone centre and is the lead in a network of radiotherapy services nationwide.

The survey was distributed [March-July 2021] to the 9 centres, after obtaining approval from each audit committee [2021]. *Qualtrics* (Qualtrics, n.d.) was used, and a link was circulated to one key person in each hospital, usually a SC advisor or member of the health promotion staff, to complete the survey, with engagement from other relevant staff as appropriate. The incentive was to contribute to a national study and to receive the study results.

SC information included (i) recording of smoking status, (ii) strategies used to support quit attempts (iii) advice provided on SC supports available within the hospital and in the community; (iv) SC programme available in-hospital (to include any behavioural or pharmacological support; brief intervention, ongoing or intensive SC support, written materials, referral to more intensive specialist supports, and availability of intensive supports onsite). The information gathered was anonymised; MS Excel was used for descriptive analysis.

## 3 Results

The response rate was 88.9% with data reported from seven specialist adult cancer hospitals and one specialist radiotherapy centre, all of whom indicated that they had some SC related provision (100%). One hospital could not identify an appropriate person to complete the survey. Another hospital found it difficult to complete the survey in full due to a vacant post, which resulted in the lack of provision of an active in-hospital SC service.

More than half (5) of the hospitals reported that all (3) or some (2) patients overall were asked about their smoking and provided with SC advice. Most hospitals (7) reported some level of SC support or service provision, to all (3) or to most (4) patients. While the data on SC services for overall patients was relatively complete and many hospitals were doing well against the guidelines, this was not the case for patients with cancer, with limited detailed data available by diagnosis and anecdotal evidence of limited referrals for cancer patients.

In seven hospitals, the service was available for general patients in all (3) or some (4) areas: *Inpatient* (*admission, during hospitalisation, at discharge), outpatients, other visits (attending radiology department, emergency department or other)*. Some form of SC service or support was provided by all of the following professionals *(medical staff, nursing staff, hospital SC staff, community SC staff (for patients referred on discharge), other allied healthcare professionals, other)* in four hospitals, while in three it was provided by some of these personnel. When asked if all patients are routinely asked about smoking, three said yes, one said yes except oncology patients, one said yes except outpatients, one said no, one was unsure and two gave no response. There was a lack of information provision across hospitals on the harms of e-cig/vaping use, although use in hospitals is banned.

Table 1 presents the results regarding provision of smoking cessation services in specialist hospitals for smokers with cancer. Stop smoking medications were provided to cancer inpatients in two hospitals, and to cancer patients at outpatients and for patients attending day ward services in one hospital. Smokers with cancer were referred automatically to the SC service in two hospitals at diagnosis. This corresponded with commencing chemotherapy treatment and in the second hospital commencement of radiotherapy. While stop smoking medications were available 24 hours a day in five hospitals, most hospitals did not stock all three stop smoking medications (NRT, Bupropion, Varenicline). One hospital advised they had data on uptake of SC services for smokers with cancer, but they were unable to identify the percentage uptake. Overall, five hospitals had staff who completed Making Every Contact Count (MECC) training programme provided by the Irish Health Service Executive for all HCPs to complete (Health Service Executive, n.d.).

**Table 1:** Description of provision of smoking cessation services in specialist hospitals for adult smokers with cancer, Ireland, 2021.

Questions	Options	N (%)
Occasions SC services provided to patients with cancer	Oncology OPD Oncology inpatients Oncology Day Patients Cancer patients attending radiology/emergency etc.	4 (44.4) 4 (44.4) 4 (44.4) 1 (11.1)

Availability of SC medications in the hospital	24/7 <24/7 Not answered	5 (55.6) 3 (33.3) 1 (11.1)

Stop smoking medications routinely provided for	Outpatients (oncology) Inpatients (oncology) Day ward (oncology)	1 (11.1) 2 (22.2) 1 (11.1)

Automatic referral of patients with cancer to SC service	At diagnosis When commencing systemic treatment When commencing radiotherapy Don’t know	1 (11.1) 1 (11.1) 1 (11.1) 4 (44.4)

Data available on uptake of SC services for patients with cancer	Inpatients Day patients	1 (11.1) 1 (11.1)

Percentage uptake of SC services by patients with cancer	Not asked* Unsure Not answered	6 (66.6) 1 (11.1) 1 (11.1)

Data available on which staff have received SC training	All staff Specific groups None No response	2 (22.2) 2 (22.2) 3 (33.3) 2 (22.2)

Provision of stop smoking medications NRT, Bupropion & Varenicline	All three products NRT & Bupropion NRT & Varenicline NRT only	2 (22.2) 0 (33.3) 4 (44.4) 1 (11.1)

Hospitals have data on SC training received by HCPs including oncology	Making every contact count (MECC) Brief intervention training Motivational interviewing Other	5 (55.6) 1 (11.1) 1 (11.1) 2 (22.2)

* Not asked as answered *no* to question above.

Table 2 shows the proportion of discharge episodes where current smoking is recorded from hospitals included in the study, both overall patients and among patients with cancer only, as collated on a regular basis by the Hospital Inpatient Enquiry (HIPE), part of the Health Service Executive in Ireland.

**Table 2:** Proportion of discharge episodes where current smoking is recorded from hospitals included in the audit (adult patients in specialist cancer hospitals in Ireland 2019).

Hospital	Overall proportion of discharge episodes where current smoking is recorded	Patients with cancer only: Proportion of discharge episodes where current smoking is recorded
1	4.7%	7.6%
2	4.7%	2.8%
3	4.9%	1.9%
4	5.7%	3.0%
5	7.3%	8.3%
6	3.5%	3.4%
7	5.5%	5.6%
8	5.4%	5.4%
9	13.4%	13.5%

## 4 Discussion

The results of this audit confirm a suboptimal, non-uniform provision of SC services provided to smokers with cancer. This audit shows that few hospitals ask all patients about smoking and offer SC services, while others asked and offered SC services to only some patients. Some of the SC offered included advice, medication, support and access to an intensive service. One hospital reported recording all relevant smoking data and 55.5% (5/9) of hospitals had data on the uptake of specialist SC services for all patients.

The new clinical guidance recommends that varenicline, alone or in combination with NRT, is offered as a first-line treatment, and if for some reason varenicline is contraindicated, combination NRT should be offered. NRT monotherapy, or bupropion (alone or in combination with NRT) or nortriptyline can also be recommended as a second-line therapy. Our audit did not seek information on nortriptyline as it was not at that time a recommended therapy. Just two hospitals audited offered the provision of all other recommended stop smoking medications while half of hospitals advised that bupropion was not available, and none reported that stop smoking medications were routinely provided to all patients. It is clear that hospitals who participated in this audit are not fully compliant with the National Clinical Guideline for SC. This may be somewhat expected as the audit was completed just before the national clinical guideline was published, however the value of smoking cessation to patients with cancer is well known.

This study is part of a wider programme of research looking at the feasibility of developing a SC pathway which includes qualitative interviews with patients and oncology HCPs. In the majority of oncology centres included in this report, the oncology service line was embedded within a larger health care system; as part of our audit we looked at the smoking cessation service across the whole hospital, but as there is specific concern about oncology patients the service for these patients was specifically reviewed.

The new national clinical guidance (Department of Health, 2022) has three clear recommendations that directly relate to the general adult population. Firstly, all HCPs should ask about an individual’s smoking behaviour. Secondly, all HCPs should advise all smokers about the harms of smoking for themselves and others and the benefit of quitting, and they should advise that help can be provided or arranged to support a quit attempt. Where a patient is interested in quitting, treatment needs, and preferences should be discussed. HCPs should advise that making an unsupported quit attempt is less effective than using recommended supports, and treatment should be provided or arranged. Thirdly, for people who are currently interested in quitting, all HCPs should recommend that behavioural support, either alone or in combination with pharmacological supports, increases the chances of successful quitting. Smoking status and all discussions, interventions and outcomes should be documented.

There is considerable variation in the smoking rates across hospitals. This reflects three current challenges in accuracy of smoking rates in hospitals in Ireland; the under ascertainment of smoking rates for all patients, which is a feature both nationally (Fitzpatrick et al., 2022) and internationally (Hirvonen et al., 2021); the variation in the prevalence of smoking in the catchment areas of the hospitals, with smoking rates higher in more deprived areas (Department of Health. Healthy Ireland Survey, 2021); and the fact that the data in Table 2 comes from a hospital discharge data system and each episode is counted, hence those hospitals with radiotherapy may appear to have higher rates due to repeated episodes.

An audit of websites of 62 National Cancer Institute (NCI) designated cancer centres (DCCs) was performed to identify institutions with online evidence of a systemwide tobacco treatment programmes (TTPs) servicing cancer patients (Day et al., 2019). The audit identified 47 NCI-DCCs TTPs. Seventeen TTPs were housed within the cancer centre and 30 TTPs were offered by the primary affiliated institution; among the latter group, only 13 TTPs were identifiable via the NCI-DCC webpage. Just three reported systemwide TTP outcomes.

Despite supportive policies from cancer organisations, treatment for tobacco dependence is still not part of standard cancer care (Morgan et al., 2011), and only 7% of cancer clinical trials assess smoking status (Gregorio et al., n.d.). Barriers to the provision of tobacco dependence treatment in the cancer context are common across health care settings (e.g., lack of physician time, concerns around appearing judgmental or eliciting feelings of guilt among patients). This is a particular challenge for people who smoke and develop cancer, for whom a combination of individual and system factors mean that their smoking cessation needs are often not consistently and fully met during cancer care (Papadakis et al., 2018, McPhee and Detmer, 1993).

McPhee et al noted physician forgetfulness, patient refusal, and practice logistical difficulties are among the major reasons that physicians perform cancer prevention activities less frequently than recommended by established guidelines in the United States (US) (McPhee and Detmer, 1993). They found that office systems are effective strategies in promoting cancer prevention activities including in-reach medical record checklists and flow sheets, stickers and alerts, audit with feedback, nurse-initiated reminders, and computer-generated reminders and outreach reminder postcards, letters, telephone calls, and questionnaires. The authors review the literature supporting the efficacy of such office systems.

This audit is the first of its kind published in Ireland whereas the UK has previously audited the provision of hospital-based SC services. (Proctor et al., 2013) Proctor *et al*, similar to the current audit, found that there was considerable variation in how SC services were staffed and run (Proctor et al., 2013). All of the inpatient wards were able to provide NRT and just over half offered varenicline and a third bupropion. These findings were supported by a British Thoracic Society (BTS) audit of UK hospital SC services in 2019 against National Institute for Health and Care Excellence and BTS standards, noting adherence to these national standards was low (Mangera and Devani, 2020). Neither of these UK studies reported electronic cigarettes information provision, while our current audit showed there was a lack of information provision across hospitals on the harms of e-cig/vaping use.

What are the solutions to the suboptimal smoking cessation services for patients with cancer? Warren et al in a study of US clinicians working in oncology found that the key barriers to providing cessation support were a lack of clinician education or experience and lack of available resources to refer patients for smoking cessation support (Warren et al., 2015). Ensuring a comprehensive smoking cessation service in each specialist cancer hospital is essential. Embedding of a liaison smoking cessation expert within the oncology service and ensuring all oncology staff take the MECC training which incorporates brief intervention training (Health Service Executive, n.d.) would improve the service without considerable resource implications.

This audit was undertaken in response to the clinical guidelines for smoking cessation published. This work is part of a wider programme of research in the area of smoking cessation for cancer patients. In this further research we have interviewed both patients and health care professionals working in oncology about their own experiences of smoking cessation for patients with cancer.

We have shown considerable variation in the SC information and services provided to cancer patients across the adult cancer specialist centres in Ireland. Audits such as this are essential to demonstrate gaps in service and provide a baseline for improvement.

## Declaration of Competing Interest

The authors declare that they have no known competing financial interests or personal relationships that could have appeared to influence the work reported in this paper.

## Data Availability

Data will be made available on request.
